# A genome wide association study for the number of animals born dead in domestic pigs

**DOI:** 10.1186/s12863-018-0692-x

**Published:** 2019-01-07

**Authors:** Pingxian Wu, Kai Wang, Jie Zhou, Qiang Yang, Xidi Yang, Anan Jiang, Yanzhi Jiang, Mingzhou Li, Li Zhu, Lin Bai, Xuewei Li, Guoqing Tang

**Affiliations:** 1 College of Animal Science and Technology, Sichuan Agricultural University, Chengdu, Sichuan China; 2 College of Life Science, Sichuan Agricultural University, Yaan, Sichuan China

**Keywords:** GWAS, Mummified pig, Pig, Stillbirth, Sow parity

## Abstract

**Background:**

The number of animals born dead, which includes the number of mummified (NM) and stillborn (NS) animals, is the most important trait to directly quantify the reproductive loss in domestic pigs. In this study, 282 Landrace sows and 250 Large White sows were genotyped by sequencing (GBS). A total of 816 and 1068 litter records for NM and NS were collected from them. A genome-wide association study (GWAS) was conducted to reveal the genetic difference between NM and NS.

**Results:**

A total of 248 and 10 genome-wide significant SNPs were detected for NM and NS across numerous parities in Landrace pigs. The corresponding numbers for Large White pigs were 175 and 6, respectively. All of the detected SNPs were parity specific for both NM and NS in two breeds. Based on significant SNPs, in total 242 (146 for Landrace pig, 96 for Large White pig) and 10 significant chromosome regions (8 for Landrace pigs, 2 for Large White pigs) were found for NM and NS, respectively. Among them, 237 (142 for Landrace pig, 95 for Large White pig) and 8 significant chromosome regions (6 for Landrace pigs, 2 for Large White pigs) for NM and NS were not reported in previous studies. A list of candidate genes at the identified loci was proposed, including HMGB1, SOX5, KCNJ8, ABCC9 and YY1 for NM, ASTN1 for NS.

**Conclusion:**

This is the first time when GBS data was used to identify genetic regions affecting NM and NS in Landrace and Large White pigs. Many identified informative SNPs and candidate genes advance our understanding of the genetic architecture of NM and NS in pigs. However, further studies are needed to validate using larger populations with more breeds.

**Electronic supplementary material:**

The online version of this article (10.1186/s12863-018-0692-x) contains supplementary material, which is available to authorized users.

## Background

Sow reproductive performance is one of the most important production traits in the pig industry. The total number born (TNB) and the number born alive (NBA) are two important traits to measure sow reproductive performance. The difference between them is defined as the number of piglets born dead, which directly measures loss in litters from the foetal stage to farrowing. The number born dead includes the number mummified (died before farrowing) (NM) and stillborn (dies during or shortly before farrowing) (NS). NM and NS are the most important traits for quantifying the loss of reproduction. In a normal clean environment, mummified piglets usually coincide with large litters, an insufficient nutrient supply, delayed placental development [1] and genetic defects [2]. Stillbirths are usually related to large litters, older sow age, slow farrowing or farrowing difficulties [1].

The basis of litter loss is multifactorial due to the quantitative characteristics of the traits, variation is determined by many genes with mostly small effects and an unknown number of non-genetic effects. Using molecular markers, many linkage studies have been conducted to find QTL and causal genes for NM and NS [3]. In the past few years, with the availability of high throughput genotyping, genome-wide association studies (GWAS) have been developed to identify DNA variants that are associated with complex diseases and genetic traits in humans and animals [4]. GWAS can be used to study the genetic architecture of swine reproductive loss. Using SNP chip information, many GWAS have been implemented to find QTL and causal mutations for NM and NS [3]. Recently, the availability of genome sequence information enabled to study the genomic architecture of complex quantitative traits, and several GWAS have been conducted based on whole genome sequence data [5]. However, there are limitations on quantitative trait studies due to the very large number of samples required, and these limitations have resulted in a very large cost for genotyping in sequence-based GWAS. Reduced-representation sequencing methods that use restriction enzymes for digestion to reduce genome complexity were found to be suitable for detecting SNPs from large numbers of samples with high reproducibility and low sample costs [6]. Genotyping by sequencing (GBS) is one such highly efficient technology for SNP detection on a genome-wide scale. This process has been successfully applied to plants and animals [3].

In general, the parity of sows significantly affects the sow’s reproduction performance because it reflects the sow’s reproductive age and physiological status. At prime parity, the litter size is smallest partly due to physiological immaturity. Up to the parity 4, 5, and 6, the litter size rapidly increases and then tends to stabilize, but it begins to decrease around parity 8 [7]. However, very little is known about the genetic architecture controlling the number of animals that are born dead in a time series focused on sow parity. In addition, the differences in genetic architecture between NM and NS for different parities are also poorly understood. Therefore, the research objective of this study was to identify significant chromosome regions that affect NM and NS in numerous parities and generate a list of candidate genes for the identified significant chromosome regions. The findings of this study provide new insight into the genetic mechanism of the number of animals that are born dead in domestic pigs.

## Methods

### Populations and phenotypes

Two pig breeds populations were used in this study: one Large White and one Landrace pig population. Two populations derived from the same commercial pig farm (Sichuan Tianzow Breeding Technology Co., Ltd), and introduced from Canadian Hylife Company at 2008. A total of 282 Landrace and 250 Large White producing sows were sampled randomly, and the pedigree data include 520 Landrace and 758 Large White pigs in two generations. In total, 816 and 1068 litter records for NM and NS were collected from the database of pig farm in 2012–2015, respectively. These records were grouped according to sow parity. For the Landrace pigs, the litter numbers were 282, 234, 179 and 121 for parities 1, 2, 3 and 4, respectively. For Large White pigs, the litter numbers were 250, 249, 244, 208 and 117 for parities 1, 2, 3, 4 and 5, respectively. Details are listed in Table 1.

**Table 1:** Observation number, mean, standard deviation, minimum and maximum of NM and NS for Landrace and Large White

Breed	Parity	Trait	N	Mean	SD	Minimum	Maximum
Landrace	1	NM	282	0.245	0.648	0	6
	1	NS	282	0.911	1.283	0	8
	2	NM	234	0.179	0.650	0	7
	2	NS	234	0.509	0.845	0	5
	3	NM	179	0.268	0.915	0	10
	3	NS	179	0.933	1.516	0	11
	4	NM	121	0.198	0.476	0	2
	4	NS	121	0.727	1.033	0	6
Large White	1	NM	250	0.184	0.497	0	3
	1	NS	250	1.148	1.385	0	6
	2	NM	249	0.213	0.553	0	3
	2	NS	249	1.032	1.439	0	9
	3	NM	244	0.385	1.370	0	14
	3	NS	244	1.402	1.494	0	7
	4	NM	208	0.173	0.500	0	3
	4	NS	208	1.563	2.006	0	14
	5	NM	117	0.256	0.800	0	7
	5	NS	117	1.607	1.965	0	10

### Genotypes and quality control

A total of 250 Large White and 282 Landrace sows were genotyped using GBS technology (Novegene limited Inc.). Genomic DNA was extracted from ear tissue using a surfactant and the protease pyrolysis method. Genomic DNA samples were checked using agarose gel electrophoresis, Nanodrop and Qubit. Then, 0.1–1 μg genomic DNA was digested with the restriction endonuclease *Mse*I. Next, P1 and P2 barcode adapters that recognize Mse1-compatible sequences were ligated to the digested DNA fragments. The restriction fragments were enriched by PCR amplification with adapter-specific primers. The quality evaluation was performed using Qubit2.0, Agilent 2100 and Q-PCR. The data sequence from the pair-end reads were generated with the Illumina HiSeq PE150. In the raw reads, N contents with > 10% of sequence length or with low quality bases (< 5) and a number > 50% of the sequence length were removed. Then, barcode sequences were eliminated. The clean data were aligned to the pig reference genome (Sscrofa11.1, Ensembl) using Burrows-Wheeler Aligner (BWA) with the parameters mem -t 4 -k 32 -M [8]. The genome analysis toolkit GATK was used to detect the SNPs using a Bayesian model [9]. Initially, a total of 10,445,924 SNPs were found. Quality control was implemented by VCFtools [10] with a minor allele frequency (MAF) of 0.01, missing rate of 0.2, Hardy-Weinberg equilibrium of 10^− 5^, and sites with a mean depth greater than or equal to 3 (dp3). After quality control, a total of 345,570 SNP markers met the quality requirements.

### Statistical analysis

For GWAS, single-marker regression analysis was conducted using GEMMA software [9]. Because the phenotypic distributions for NM and NS were not normal, a transform $\sqrt{x+1}$ for all of phenotypes were conducted firstly, where *x* is the phenotype value. Then, the birth year and month are included as a fixed effect, and the DMU software [11] was used to adjust these phenotype value. A univariate mixed linear model was utilized to evaluate the association between qualified SNPs and phenotypic values, separately for NM and NS in each breed and each parity.

$$ \mathbf{y}=\mathbf{Z}\boldsymbol{\upbeta } +\mathbf{Wa}+\mathbf{e}, $$

where **y** is the vector of phenotypic observation; *β* is the SNP effect; **a** is the vector of the remaining polygene effect following the multi-normal distribution ($\mathrm{a}\sim \mathrm{MVN}\left(0,\mathrm{A}{\sigma}_a^2\right)$), A is a numerator relationship matrix; **e** is the vector of residual effects following the multi-normal distribution ($\mathrm{e}\sim \mathrm{MVN}\left(0,\mathrm{I}{\sigma}_e^2\right)$); **Z**, **W** are incidence matrices for *β* and **a**, respectively. Bonferroni correction methods were used to adjust *P* value in multiple testing. Using the Bonferroni method, a genome level (0.05/N) and a suggestive (1/N) thresholds were used in this study, where N is the number of SNPs used for analyses. In Landrace, a total of 251,678, 247,225, 245,353 and 240,368 SNPs for parity 1–4 were used to analysis. In Large White pigs, a total of 277,080, 275,687, 274,867, 272,405 and 271,736 SNPs for parity 1–5 were used to analysis. The percentage of phenotypic variance explained by each top significant SNP was calculated by ${\sigma}_r^2-{\sigma}_f^2/{\sigma}_r^2$, where ${\sigma}_f^2$ and ${\sigma}_r^2$ are residual variances of linear models with and without SNP genotypes as predictor variables, respectively.

### LD analysis

To detect the linkage disequilibrium (LD) between significant SNPs, the 40Kb region centring on each top SNP (with the highest –log(P) value) was used to performed LD analysis by Haploview software [12].

### Candidate gene search

To identify candidate genes for genome-wide significant loci, a search for annotated genes within a 40Kb region centring on each top SNP (with the highest –log(P) value) of significant loci in the pig reference genome assembly (Build 11.1) was implemented using ANNOVAR software [13].

## Results

### Single-parity GWAS for NM in Landrace pig population

**Fig. 1:**
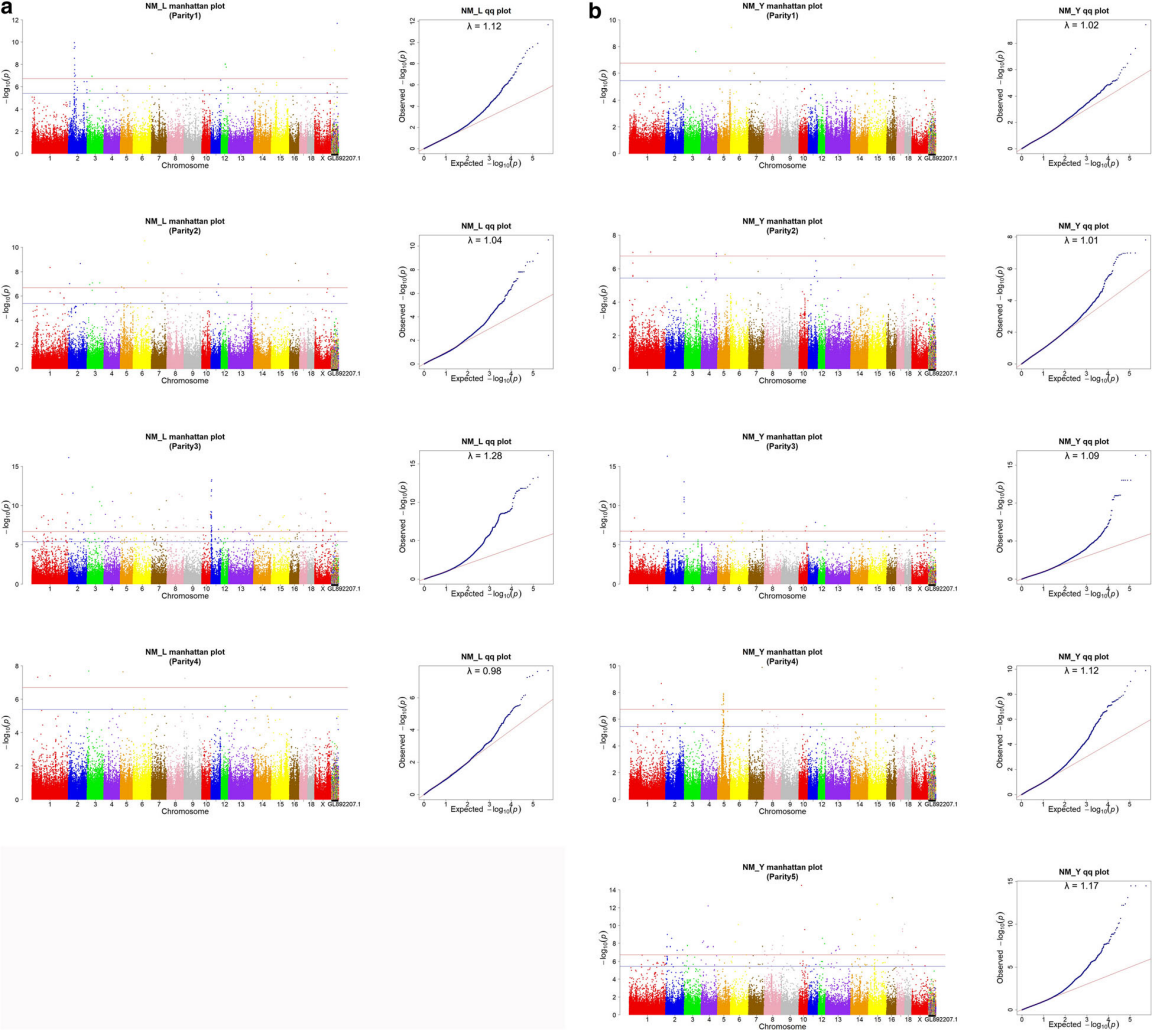
Manhattan and QQ plot of genome-wide association for number mummified (NM) in Landrace pig (**a**) and Large White pig (**b**) population. The light red line represents the genome level significance threshold based on 0.05/N, the light blue line represents the suggested level significance threshold based on 1/N, where N is the number of SNPs used for analyses. The lambda is the genomic inflation factor

In each parity, a mixed linear model was used to implement a single SNP association test. The association patterns of SNPs with NM in Landrace pigs are shown in Table 2 and Fig. 1a (left side). In this study, two adjacent significant SNPs were merged together if their distance was smaller than 40Kb. Table 2 only lists those significant chromosome regions that are genome level significant (0.05/N) and the SNP number involved is at least larger than 2. All detected genome-wide significant SNPs are listed in Additional file 1: Table S1.

**Table 2:** Summary of significant chromosome regions for number mummified (NM) at genomic level in Landrace population

Parity	SSC	Range (Mb)	Number	Position(bp)	Alleles	*P* value	Candidate Gene
1	2	51.85–55.30	7	53,226,790	G/C	1.17E-10	OR2T6
1	12	37.88–37.92	2	37,895,292	C/T	9.23E-09	
1	12	45.64–45.68	2	45,662,205	T/C	1.84E-08	TAOK1
2	11	64.1–64.17	2	64,146,608	C/T	1.03E-07	ABCC4
2	X	112.6–112.64	6	112,615,739	C/G	1.51E-08	ZIC3
3	1	141.79–141.83	2	141,813,666	T/A	2.47E-08	
3	1	295.95–295.99	6	295,973,126	G/A	1.59E-08	
3	2	40.27–40.31	2	40,285,249	T/C	2.61E-12	ZDHHC13
3	10	73.77–73.81	4	73,789,449	C/T	1.42E-09	
3	11	1.28–7.13	99	7,114,076	A/G	5.25E-14	HMGB1/CDK8/SAP18/SKA3/MIPEP/SPATA13/ MTMR6/NUP58/ATP8A2
3	X	69.58–69.62	4	69,596,000	G/A	1.41E-07	

*Range* Range of significant chromosome region, *Number* Number of SNP involved, *Position* Position of top SNP, *Alleles* Alleles of top SNP, *Candidate Gene* Gene found in the range

Three peaks were observed from Fig. 1a. The most significant chromosome region was located in 1.28–7.13 Mb on SSC11 in parity 3 and its top SNP (Position: 7114076 bp; *P* = 5.25E-14) was located in HMGB1 gene, which explained 1.36% of the phenotypic variance. In this region, a total of 99 SNPs were detected and 8 positional candidate genes was located (Table 2). The second significant chromosome region was in 51.85–55.30 Mb on SSC2 in parity 1. In this region, a total of 7 SNPs was detected and only a candidate gene OR2T6 was determined. The number of significant SNP detected in parity 3 is the most, then is parity 1, and the least is parity 4 (Additional file 1: Table S1).

### Single-parity GWAS for NM in large white pig population

The association results of SNPs with NM in Large White pigs based on single SNP tests are shown in Table 3 (only including the significant chromosome region with SNP number larger than 2) and Fig. 1b (right side). All detected genome-wide significant SNPs are listed in Additional file 1: Table S2.

**Table 3:** Summary of significant chromosome regions for number mummified (NM) at genomic level in Large White population

Parity	SSC	Range (Mb)	Number	Position(bp)	Alleles	P value	Candidate Gene
2	1	32.30–32.34	2	32,322,537	A/G	1.10E-07	AKAP7
2	1	185.49–185.53	3	185,506,783	C/A	1.04E-07	
2	4	133.07–133.11	2	133,090,486	A/G	1.90E-07	
3	2	159.12–159.35	13	159,334,109	C/T	9.58E-14	
3	18	15.29–15.42	8	15,306,205	A/G	1.05E-11	EXOC4
4	2	53.53–53.57	5	53,550,664	A/T	8.13E-08	
4	5	36.73–36.77	5	36,749,866	T/G	8.50E-08	
4	5	49.74–53.88	28	49,763,855	A/G	2.89E-08	SOX5/ST8SIA1/KCNJ8/ABCC9/GYS2/SPX/GOLT1B/PLEKHA5
4	7	116.70–116.74	2	116,719,034	G/T	1.32E-10	SYNE3
4	15	62.98–63.02	14	63,000,645	A/T	9.45E-10	
5	2	14.95–14.99	7	14,966,625	G/A	1.06E-09	AGBL2/MTCH2
5	2	52.21–52.25	2	52,227,328	T/C	2.76E-09	LOC100525099
5	3	27.64–27.90	4	27,657,943	T/A	1.68E-08	
5	4	62.24–62.28	2	62,259,407	A/C	6.18E-13	STAU2
5	7	121.12–121.16	5	121,138,988	C/G	2.16E-08	YY1
5	8	5.54–5.58	2	5,558,816	A/G	6.84E-08	
5	10	21.60–21.64	3	21,623,235	C/T	3.25E-15	
5	15	52.89–52.93	4	52,912,314	T/C	1.44E-09	
5	17	50.86–50.90	2	50,880,718	T/G	2.49E-10	STAU1

*Range* Range of significant chromosome region, *Number* Number of SNP involved, *Position* Position of top SNP, *Alleles* Alleles of top SNP, *Candidate Gene* Gene found in the range

Four peaks were observed from Fig. 1b. The first significant chromosome region was in 49.74–52.74 Mb on SSC5 in parity 4 and its top SNP was located in SOX5 gene, which explained 2.26% of the phenotypic variance. In this region, a total of 28 SNPs were detected and a list of candidate genes was located, including SOX5, ST8SIA1, KCNJ8, ABCC9, GYS2, SPX, GOLT1B and PLEKHA5. The second significant chromosome region was located in 62.98–63.02 Mb on SSC15 in parity 4 and its number of SNP involved was 14. However, in this region, no candidate gene was found. The third significant chromosome region was located in 159.12–159.35 Mb on SSC2 in parity 3. A total of 13 SNPs were detected in this region, but no candidate gene was located. The fourth significant chromosome region was located in 121.12–121.16 Mb on SSC7 in parity 5. A total of 5 SNPs were detected in this region and a candidate gene YY1 was found here. The association patterns of SNPs for NM in Large White pigs across five parities were distinctly different with Landrace pigs. The number of detected significant SNPs in parity 5 is the most, the latter is parity 4, and the least is parity 1 (Additional file 1: Table S2).

### Single-parity GWAS for NS

**Fig. 2:**
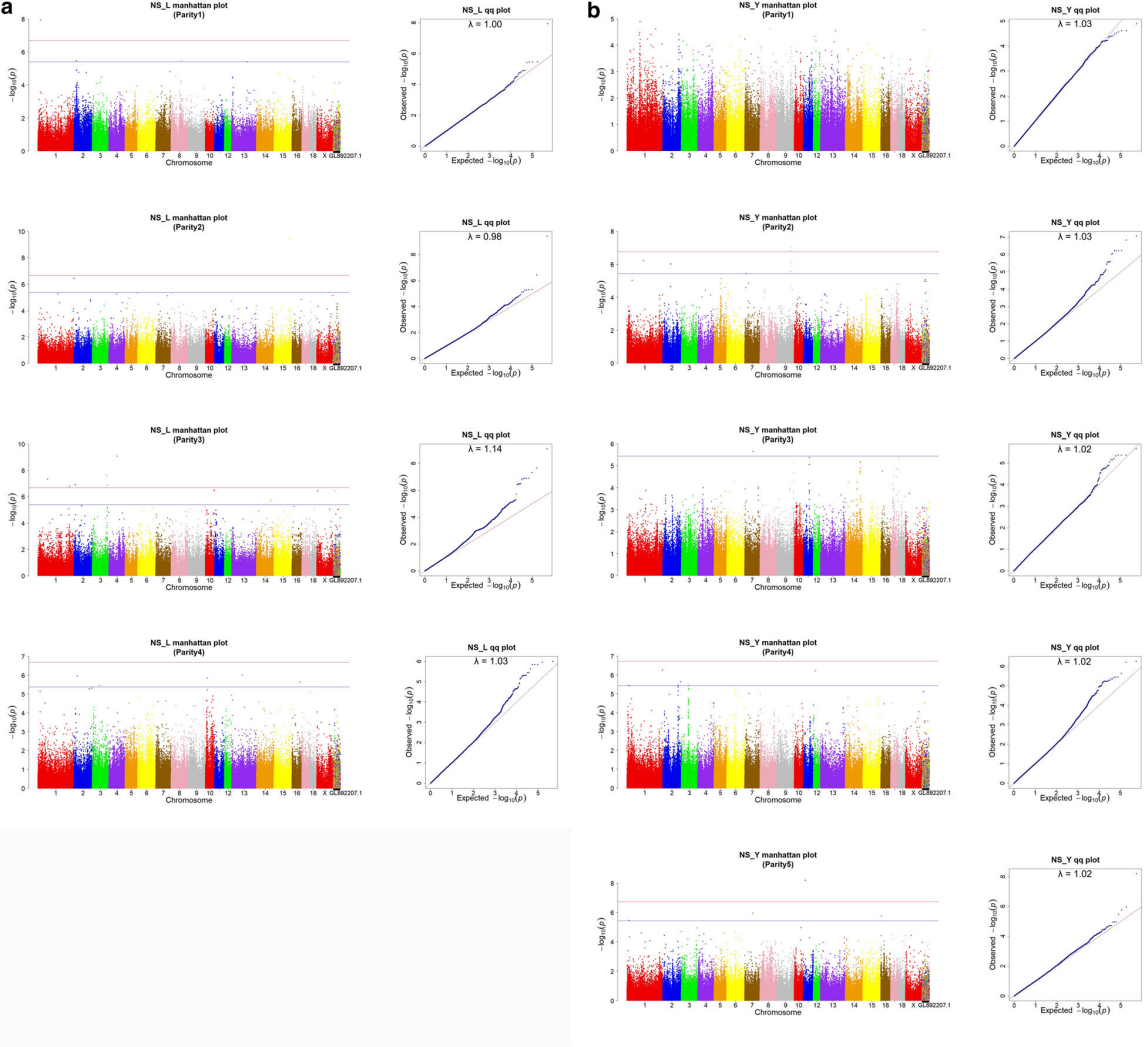
Manhattan and QQ plot of genome-wide association for number stillborn (NS) in Landrace pig (**a**) and Large White pig (**b**) population. The light red line represents the genome level significance threshold based on 0.05/N, the light blue line represents the suggested level significance threshold based on 1/N, where N is the number of SNPs used for analyses. The lambda is the genomic inflation factor

The association results of SNPs with NS in Landrace and Large White pigs are shown in Table 4 and Fig. 2. Table 4 only lists one genome level significant chromosome region with SNP number larger than 2 for Landrace and Large White pigs. Other significant SNPs and significant chromosome regions for NS are listed in Additional file 1: Tables S3 and S4. All of these detected significant chromosome regions for NS in this study were parity-specific (Additional file 1: Tables S3 and S4).

**Table 4:** Summary of significant chromosome regions for number stillborn (NS) at genomic level

Breed	Parity	SSC	Range (Mb)	Number	Position(bp)	Alleles	P value	Candidate Gene
Landrace	3	3	131.82–131.86	3	131,841,442	G/A	1.29E-07	
Large White	2	9	118.97–119.18	8	118,987,531	G/A	8.89E-08	ASTN1/BRINP2

*Range* Range of significant chromosome region, *Number* Number of SNP involved, *Position* Position of top SNP, *Alleles* Alleles of top SNP, *Candidate Gene* Gene found in the range

In Landrace pig population, one significant chromosome region was located in 131.82–131.86 Mb on SSC3 in parity 3. However, no candidate gene was found in this region. In Large White pig population, one significant chromosome region was mapped to 118.97–119.18 Mb on SSC9 in parity 2, and two candidate genes (ASTN1 and BRINP2) were located in this region.

### LD results

**Fig. 3:**
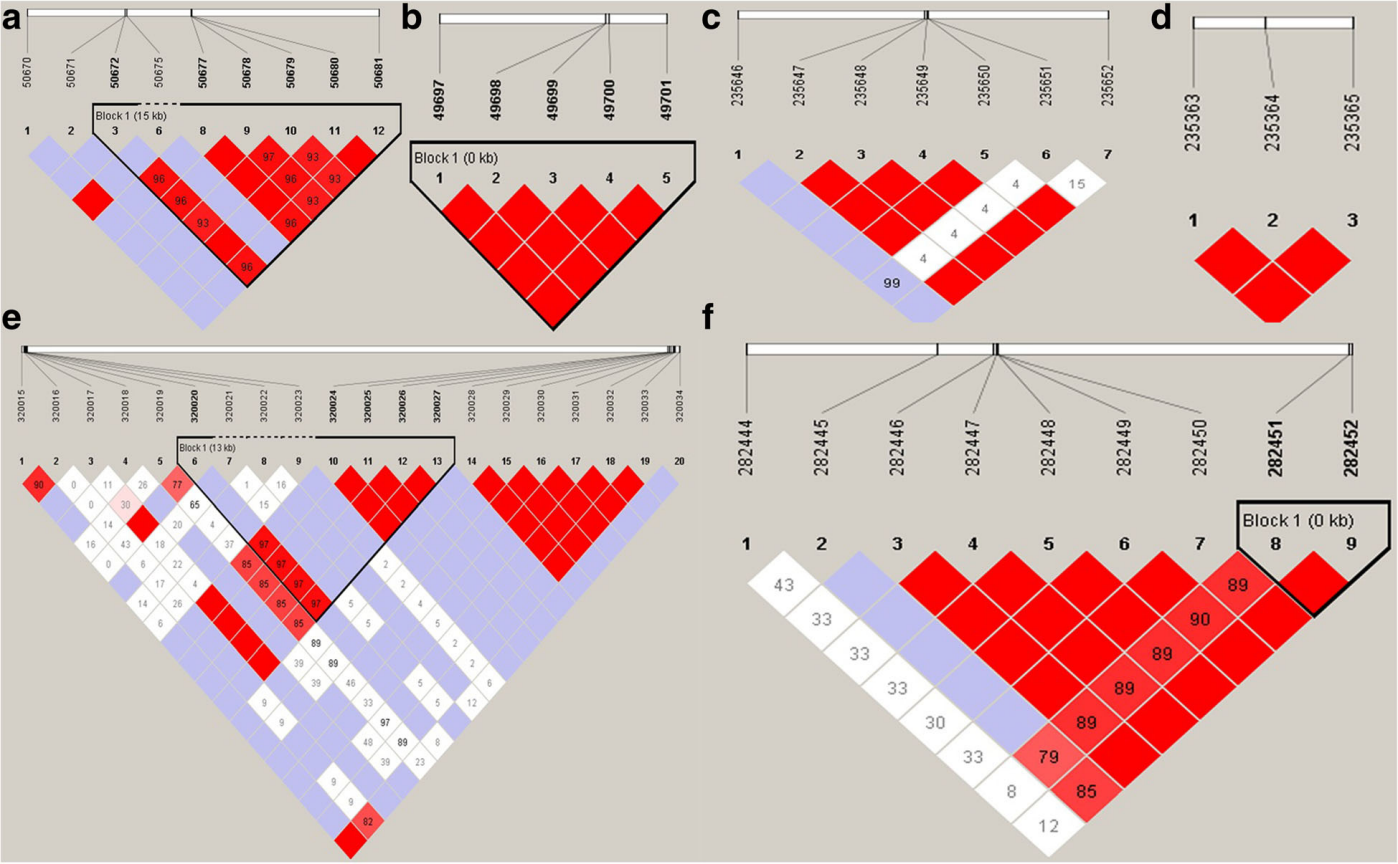
Linkage disequilibrium plots of significantly associated 40Kb regions centering on each significant SNP (SSC11: 2898066 bp (**a**); SSC11: 7114076 bp (**b**); SSC5: 51702429 bp (**c**); SSC5: 49763855 bp (**d**), SSC9: 118987531 bp (**e**), SSC7: 121138988 bp (**f**)). Values in boxes are LD (R^2^) between SNP pairs and the boxes are colored according to the standard color scheme: LOD > 2 and D’ = 1, red; LOD > 2 and D’ < 1, shades of pink/red; LOD < 2 and D’ = 1, blue; LOD < 2 and D’ < 1, white (LOD is the log of the likelihood odds ratio, a measure of confidence in the value of D’)

By carrying out associate test with GBS data, many significant SNPs were identified. This study suggested six important SNPs including SSC11: 2898066 bp, SSC11: 7114076 bp, SSC5: 51702429 bp, SSC5: 49763855 bp, SSC9: 118987531 bp and SSC7: 121138988 bp. The markers within these significant regions (40Kb region centring on each significant SNP) were used to conduct LD analysis. The LD block plots were shown in Fig. 3 for each significant region. Only one block were detected on four significant regions using confidence interval algorithm. And no LD block was found for the 40Kb region centring on two SNP (SSC5: 51702429 bp and SSC5: 49763855 bp).

## Discussion

In this study, a GWAS based on GBS data was implemented using univariate mixed linear model for animals born dead trait including NM and NS in two commercial pig populations. To eliminate the population stratification, this GWAS was performed separately in Landrace pig and Large White pig population.

### GBS technology identified novel loci for NM and NS

To the best of our knowledge, although QTL on NM and NS have been reported in previous studies [3], this was the first GWAS on NM and NS using SNP markers genotyped by GBS technology in the Large White and Landrace pig populations. For NM, in total 146 and 96 genome-wide significant chromosome regions were detected in Landrace and Large White pig population, respectively. The number of significant chromosome regions for NS was much less than NM. However, Onteru et al. (2012) [14] showed that the number of significant chromosome regions for NM and NS were (36, 25), (27, 17) and (41, 21) at parity 1, 2 and 3, respectively. The number of significant chromosome regions for NS was just slightly less than NM, moreover, most of positions of detected significant chromosome regions were different from this study. The difference may be caused by different pig populations, different genotyping methods (genotyping by sequencing vs. SNP chip) and different statistic methods (single marker regression vs. SNP sliding window). In Landrace pig population, for NM, a total of 4 significant chromosome regions overlapped with previous studies (Table 5), and the other 142 significant chromosome regions were new genomic regions that were not associated with NM before [15]; for NS, 2 significant chromosome regions overlapped with previous studies and the other 6 significant chromosome regions were new genomic region (Table 5 and Additional file 1: Table S3). In Large White pig population, only 1 significant chromosome region for NM overlapped with previous studies (Table 5) and the other 95 significant chromosome regions were new genomic region (Additional file 1: Table S2, PigQTLdb); for NS, all detected significant chromosome regions were not previously reported in literature (Table 5 and Additional file 1: Table S4). These results showed that the GBS genotyping technology can further identify novel loci relative to common commercial SNP chips. Although a lot of new SNPs for NM and NS were found in the present two populations, more validation studies are needed. In this study, the record numbers of each parity were small (see previous section), the small population may decrease the reliability of GWAS. Thus, these studies should be further verified using larger populations with more breeds.

**Table 5:** The significant chromosome regions detected in current study overlapped with previously studies from PigQTLDB (https://www.animalgenome.org)

Population	Parity	SSC	Current study			Previous studies		Trait
			Position1	Position2	*P* value	Position 1	Position 2	
Landrace	1	13	36,471,798	36,511,798	1.57E-06	36,281,164	36,541,751	mummified pigs [7]
	2	6	107,770,192	107,810,192	5.75E-08	107,747,013	125,323,355	mummified pigs [7]
	3	6	110,794,277	110,834,277	6.71E-07	107,747,013	125,323,355	mummified pigs [7]
	3	16	70,323,327	70,363,327	1.74E-06	70,336,298	70,524,616	mummified pigs [7]
	3	4	73,533,176	73,573,176	8.16E-10	73,460,141	73,747,755	number of stillborn [7]
	4	13	92,450,305	92,490,305	9.81E-07	17,040,484	120,146,164	number of stillborn [7]
Large White	5	9	10,432,963	10,472,963	1.76E-08	10,147,197	10,497,473	mummified pigs [7]

### GWAS on NM and NS to reveal the differences in genetic architecture between them

The increase in number of mummified pigs and stillbirth animals would reduce the number of animals born alive in pig production. The increase in number of mummified pigs and stillbirth animals would reduce the number of animals born alive, affecting production. However, by conducting GWAS for NM and NS, very few common significant SNPs were found. Three SNPs (SSC1, position = 85,721,479, P_NM_ = 2.99E-09, P_NS_ = 4.70E-08; SSC3, position = 129,076,023, P_NM_ = 3.96E-06, P_NS_ = 2.27E-08; SSC4, position = 73,553,176, P_NM_ = 1.30E-06, P_NS_ = 8.16E-10) were found in common between NM and NS in Landrace pig population at parity 3 (Table 6). These results implied that the two traits have very different genetic architecture and are controlled by highly polygenetic traits. In general, a few mummified piglets or stillborn occurring is not caused by disease. In this study, most NM and NS was in the range of 0–3 (see the means in Table 1). Therefore, the variation for NM and NS were caused by sow’s genetic characteristics. The GWAS results further indicated the genetic differences and different selection strategies between NM and NS.

**Table 6:** Number of significant SNP common between NM and NS for Landrace and Large White pig population

Trait1	Trait2	Number
Landrace NM	Large White NM	1
Landrace NS	Large White NS	0
Landrace NM	Landrace NS	3
Large White NM	Large White NS	0
Landrace NM	Large White NS	0
Landrace NS	Large White NM	0
Landrace NM (parity1–4)	Landrace NM (parity1–4)	0
Landrace NS (parity1–4)	Landrace NS (parity1–4)	0
Large White NM (parity1–5)	Large White NM (parity1–5)	0
Large White NS (parity1–5)	Large White NS (parity1–5)	0

### GWAS on NM and NS to reveal the differences in genetic architecture between sow parities

In this study, a phenomenon was observed in the GWAS, i.e., all of significant SNPs for both NM and NS were parity-specific, and no significant SNPs or candidate genes were in common between parities (Table 6). This results were similar to Onteru et al. (2012) [14]. They speculated that there were possible temporal gene effects for each sow’s parity. There are different physiological characteristics in different parities of sows due to age. This result showed that the genomic structures that control NM and NS were different in different parities, and further confirm that different parities should be considered as different traits. This also verified that it was necessary to conduct GWAS in different parities.

### Plausible candidate genes at significant loci

By separately conducting GWAS for NM and NS in Large white and Landrace pig populations, several known genes that have their functions related to lethality, expression of reproductive system and embryo mesenchyme were found in this study. Thus, it is important to identify these genes.

At 1.28–7.13 Mb on SSC11, a significant chromosome region for NM was detected in Landrace pig population at parity 3 (Table 2). Two important functional candidate genes (HMGB1 and SPATA13) were located in this region. The HMGB1 (*High Mobility Group Box 1*) gene plays a role in several cellular processes, including inflammation, cell differentiation and tumor cell migration. Knockout mutations in the HMGB1 gene resulted in lethality in mice [16, 17].

At 49.74–53.88 Mb on SSC5, a significant chromosome region for NM was detected in Large White population at parity 4 (Table 3). Three positional functional candidate genes (SOX5, KCNJ8 and ABCC9) were located in this region. The SOX5 (*SRY-Box 5*) gene is expressed in the embryo mesenchyme and many important physiological system [18] that could affect the health of sow and the embryo. And mutation of SOX5 seem to result in neonatal death, and carriers of a SOX5 deletion also show several clinical features [19]. The KCNJ8 (*Potassium Voltage-Gated Channel Subfamily J Member 8*) and ABCC9 (*ATP Binding Cassette Subfamily C Member 9*) genes are related to Hypertrichotic Osteochondrodysplasia [20]. The KCNJ8 is expressed in the embryo mesenchyme and reproductive system [21], and the mutation of KCNJ8 directly affected the immune system, homeostasis and mortality [22]. This gene plays an important role in reproductive and immune system and development of embryo. On one hand, this gene could directly affect the development of embryo. On the other hand, due to the effect on sow’s health, it could indirectly result in fetal disease or death. Furthermore, the ABCC9 gene is also associated with homeostasis and mortality [23], and is involved in the body’s immune system progress [22]. Thus, these two genes were proposed as important candidate genes for NM.

At 121.12–121.16 Mb on SSC7, a significant chromosome region for NM was detected in parity 5 (Table 3). Its top SNP was located on YY1 (*YY1 Transcription Factor*) gene. This gene is a protein-coding gene and associated with peri-implantation lethality [24]. Therefore, it was proposed as candidate gene for NM.

At 118.97–119.18 Mb on SSC9, a significant chromosome region for NS was detected in parity 2 (Table 4). Its top SNP (position: 118987531 bp, *P* = 8.89E-08) was located to the ASTN1 (*Astrotactin 1*) gene. This gene is a protein-coding gene, and related to Schimke Immunoosseous Dysplasia with clinical phenotypes of growth retardation, renal failure, recurrent infections, cerebral infarcts [25].

## Conclusions

In this study, we performed a GWAS study to detect genomic regions associated with NM and NS in different parities in two pig populations. These findings advance our understanding of the genetic architecture of the number of animals born dead in domestic pigs. Most of SNP detected were different between NM and NS. All of significant SNPs for both NM and NS were parity-specific, and no candidate gene was in common between parities. GBS technology explores many new SNPs for NM and NS, however, these SNPs should be further validated using larger populations with more breeds.

## Additional file

Additional file 1: **Table S1.** Summary of significant chromosome regions including genomic level (0.05/N) for NM in Landrace population, N is the number of SNPs used for analyses. **Table S2.** Summary of significant chromosome regions including genomic level (0.05/N) for NM in Large White population, N is the number of SNPs used for analyses. **Table S3.** Summary of significant chromosome regions including genomic level (0.05/N) for NS in Landrace population, N is the number of SNPs used for analyses. **Table S4.** Summary of significant chromosome regions including genomic level (0.05/N) for NS in Large White population, N is the number of SNPs used for analyses. (DOCX 40 kb)

## Abbreviations

GBS: Genotyping by sequencing
GWAS: Genome-wide association studies
NBA: Number born alive
NM: Number of mummified animals
NS: Number of stillborn animals
QTL: Quantitative trait locus
SNP: Single nucleotide polymorphism
TNB: Total number born
