# Supplementary material for: A genome wide association study for the number of animals born dead in domestic pigs
Source: BMC Genet. 2019 Jan 7;20:4. doi: 10.1186/s12863-018-0692-x (PMC6324166; doi:10.1186/s12863-018-0692-x)
Supplement: Supplementary file 1 — Table S1. Summary of significant chromosome regions including genomic level (0.05/N) for NM in Landrace population, N is the number of SNPs used for analyses. Table S2. Summary of significant chromosome regions including genomic level (0.05/N) for NM in Large White population, N is the number of SNPs used for analyses. Table S3. Summary of significant chromosome regions including genomic level (0.05/N) for NS in Landrace population, N is the number of SNPs used for analyses. Table S4. Summary of significant chromosome regions including genomic level (0.05/N) for NS in Large White population, N is the number of SNPs used for analyses. (DOCX 40 kb) [file 12863_2018_692_MOESM1_ESM.docx]

Table S1. Summary of significant chromosome regions including genomic level (0.05/N) for NM in Landrace population, N is the number of SNPs used for analyses.

| Parity | SSC | Range (Mb) | SNP Number | Top SNP Position(bp) | Top SNP Allele | Top SNP P Value | Candidate Gene |
| --- | --- | --- | --- | --- | --- | --- | --- |
| 1 | 2 | 53.21-53.25 | 2 | 53226790 | G/C | 1.17E-10 | OR2T6 |
| 1 | 2 | 56.98-57.02 | 1 | 57002999 | G/A | 2.62E-10 |  |
| 1 | 2 | 51.29-51.33 | 1 | 51307391 | C/T | 3.71E-10 | IBA57 |
| 1 | 2 | 56.40-56.44 | 1 | 56421251 | C/T | 1.14E-08 | OR13G1 |
| 1 | 2 | 55.41-55.45 | 1 | 55427732 | A/T | 2.99E-08 |  |
| 1 | 2 | 51.85-51.89 | 3 | 51868985 | G/T | 6.16E-08 |  |
| 1 | 2 | 60.44-60.48 | 1 | 60462680 | A/G | 9.22E-08 | USE1/OCEL1/MYO9B |
| 1 | 2 | 55.26-55.30 | 2 | 55275268 | T/G | 1.21E-07 |  |
| 1 | 3 | 42.53-42.57 | 1 | 42553691 | T/A | 1.16E-07 | FGD3 |
| 1 | 3 | 43.99-44.03 | 1 | 44009233 | C/T | 1.21E-07 | TTL |
| 1 | 7 | 5.04-5.08 | 1 | 5055621 | C/T | 1.06E-09 | BMP6 |
| 1 | 12 | 37.88-37.92 | 2 | 37895292 | C/T | 9.23E-09 |  |
| 1 | 12 | 45.64-45.68 | 2 | 45662205 | T/C | 1.84E-08 | TAOK1 |
| 1 | 17 | 37.09-37.13 | 1 | 37108611 | G/T | 2.45E-09 | CBFA2T2 |
| 1 | GL893064.1 | -0.01-0.04 | 1 | 8517 | T/A | 5.56E-10 |  |
| 1 | GL895110.2 | 0.19-0.23 | 1 | 211718 | A/T | 2.09E-12 |  |
| 2 | 1 | 158.2-158.20 | 1 | 158178113 | C/T | 4.35E-09 | SERPINB5 |
| 2 | 2 | 104.8-104.84 | 1 | 104818149 | A/C | 2.18E-09 |  |
| 2 | 2 | 10.7-10.70 | 1 | 10678515 | A/C | 9.5E-08 | CD5 |
| 2 | 3 | 46.8-46.84 | 1 | 46818053 | G/A | 8.42E-08 |  |
| 2 | 3 | 108.9-108.96 | 1 | 108943667 | A/T | 8.48E-08 |  |
| 2 | 3 | 24.1-24.14 | 1 | 24116232 | T/G | 1.11E-07 | VWA3A/C16orf52 |
| 2 | 5 | 10.5-10.53 | 1 | 10511418 | G/T | 1.79E-07 | CYTH4 |
| 2 | 6 | 100.4-100.45 | 1 | 100425861 | C/T | 3.02E-11 | LAMA1 |
| 2 | 6 | 129.0-129.08 | 1 | 129057380 | G/A | 1.83E-09 | SSX2IP |
| 2 | 6 | 107.8-107.81 | 1 | 107790192 | G/A | 5.75E-08 |  |
| 2 | 8 | 130.5-130.55 | 1 | 130529459 | C/A | 1.48E-08 | HERC5/PYURF |
| 2 | 11 | 64.1-64.17 | 2 | 64146608 | C/T | 1.03E-07 | ABCC4 |
| 2 | 13 | 198.5-198.49 | 1 | 198471919 | G/A | 1.92E-07 | RUNX1 |
| 2 | 14 | 111.7-111.70 | 1 | 111678343 | G/T | 4.19E-10 |  |
| 2 | 16 | 50.7-50.71 | 1 | 50687737 | A/G | 2.06E-09 |  |
| 2 | 16 | 79.3-79.37 | 1 | 79350779 | A/T | 5.57E-08 | SLC12A7 |
| 2 | 23 | 112.6-112.64 | 6 | 112615739 | C/G | 1.51E-08 | ZIC3 |
| 3 | 1 | 260.34-260.38 | 1 | 260356464 | T/G | 3.72E-12 |  |
| 3 | 1 | 309.58-309.62 | 1 | 309599034 | C/T | 8.69E-10 |  |
| 3 | 1 | 103.03-103.07 | 1 | 103054048 | C/T | 2.08E-09 |  |
| 3 | 1 | 85.70-85.74 | 1 | 85721479 | T/C | 2.99E-09 |  |
| 3 | 1 | 178.60-178.64 | 1 | 178621801 | A/G | 5.73E-09 |  |
| 3 | 1 | 142.55-142.59 | 1 | 142573919 | T/A | 7.58E-09 |  |
| 3 | 1 | 295.95-295.99 | 6 | 295973126 | G/A | 1.59E-08 |  |
| 3 | 1 | 141.79-141.83 | 2 | 141813666 | T/A | 2.47E-08 |  |
| 3 | 1 | 40.32-40.36 | 1 | 40341795 | T/A | 8E-08 |  |
| 3 | 2 | 5.07-5.11 | 1 | 5087905 | C/A | 7.78E-17 | CABP4/GPR152/CORO1B/RPS6KB2/CARNS1/PTPRCAP |
| 3 | 2 | 40.27-40.31 | 2 | 40285249 | T/C | 2.61E-12 | ZDHHC13 |
| 3 | 2 | 97.19-97.23 | 1 | 97208416 | T/C | 7.14E-09 |  |
| 3 | 2 | 120.04-120.08 | 1 | 120063886 | A/G | 2.6E-08 |  |
| 3 | 2 | 86.91-86.95 | 1 | 86925752 | C/A | 1.81E-07 | AP3B1 |
| 3 | 3 | 47.72-47.76 | 1 | 47743966 | A/C | 4.5E-13 | SULT1C4/GCC2 |
| 3 | 3 | 109.89-109.93 | 1 | 109907288 | T/A | 3.02E-11 | ALK |
| 3 | 3 | 126.94-126.98 | 1 | 126958856 | C/T | 1.1E-10 | ASAP2 |
| 3 | 3 | 33.04-33.08 | 1 | 33059941 | G/A | 1.55E-09 | GRIN2A |
| 3 | 3 | 27.76-27.80 | 1 | 27783953 | C/A | 1.89E-08 |  |
| 3 | 4 | 76.48-76.52 | 1 | 76503066 | A/C | 3.14E-11 |  |
| 3 | 4 | 13.89-13.93 | 1 | 13906640 | T/C | 1.35E-09 |  |
| 3 | 4 | 139.97-140.01 | 1 | 139993236 | C/A | 3.38E-09 |  |
| 3 | 4 | 95.91-95.95 | 1 | 95931362 | C/A | 8.56E-09 | INTS3 |
| 3 | 4 | 100.96-101.00 | 1 | 100983594 | A/T | 5.63E-08 | NOTCH2 |
| 3 | 5 | 94.63-94.67 | 1 | 94654696 | G/T | 2.6E-12 |  |
| 3 | 5 | 106.85-106.89 | 1 | 106871417 | A/G | 2.76E-09 |  |
| 3 | 5 | 14.19-14.23 | 1 | 14208323 | T/G | 4.25E-08 |  |
| 3 | 6 | 58.53-58.57 | 1 | 58554942 | G/T | 2.1E-08 |  |
| 3 | 7 | 129.19-129.23 | 1 | 129209615 | G/T | 2.51E-11 |  |
| 3 | 7 | 68.12-68.16 | 1 | 68137837 | C/A | 3.23E-10 |  |
| 3 | 7 | 9.29-9.33 | 1 | 9308882 | A/C | 3.03E-08 |  |
| 3 | 7 | 126.00-126.04 | 1 | 126016327 | G/T | 6.18E-08 |  |
| 3 | 7 | 22.99-23.03 | 1 | 23005609 | C/A | 9.36E-08 |  |
| 3 | 8 | 106.84-106.88 | 1 | 106859874 | T/G | 3.68E-12 |  |
| 3 | 8 | 130.56-130.60 | 1 | 130579807 | C/A | 6.67E-12 | HERC5/HERC6 |
| 3 | 8 | 144.92-144.96 | 1 | 144943750 | C/A | 4.41E-09 |  |
| 3 | 8 | 6.74-6.78 | 1 | 6762262 | C/A | 9.2E-09 | CLNK |
| 3 | 8 | 143.61-143.65 | 1 | 143629229 | C/A | 3.01E-08 |  |
| 3 | 8 | 85.05-85.09 | 1 | 85071247 | C/A | 5.63E-08 |  |
| 3 | 8 | 72.87-72.91 | 1 | 72892796 | G/A | 6.25E-08 |  |
| 3 | 8 | 87.11-87.15 | 1 | 87131953 | C/A | 7.14E-08 |  |
| 3 | 9 | 119.94-119.98 | 1 | 119963461 | C/A | 6.18E-09 |  |
| 3 | 9 | 17.97-18.01 | 1 | 17990649 | G/T | 4.23E-08 | DLG2 |
| 3 | 9 | 111.82-111.86 | 1 | 111838517 | C/A | 4.98E-08 |  |
| 3 | 10 | 73.77-73.81 | 4 | 73789449 | C/T | 1.42E-09 |  |
| 3 | 10 | 13.73-13.77 | 1 | 13745612 | G/T | 6.8E-08 | EPHX1 |
| 3 | 11 | 7.09-7.13 | 2 | 7114076 | A/G | 5.25E-14 | HMGB1 |
| 3 | 11 | 4.32-4.36 | 1 | 4342081 | C/T | 7.89E-14 | WASF3 |
| 3 | 11 | 5.47-5.51 | 1 | 5488336 | T/C | 1.01E-12 | PAN3 |
| 3 | 11 | 4.04-4.08 | 11 | 4061208 | G/A | 1.52E-12 | CDK8 |
| 3 | 11 | 2.93-2.97 | 9 | 2951879 | G/T | 6.01E-12 |  |
| 3 | 11 | 1.19-1.23 | 1 | 1208824 | G/A | 5.89E-10 | XPO4/LATS2 |
| 3 | 11 | 5.65-5.69 | 1 | 5671102 | T/C | 7.36E-10 | FLT1 |
| 3 | 11 | 2.61-2.65 | 5 | 2633207 | G/C | 1.54E-09 |  |
| 3 | 11 | 2.88-2.92 | 4 | 2898066 | C/T | 2.11E-09 | SPATA13 |
| 3 | 11 | 2.56-2.60 | 1 | 2578634 | C/T | 2.15E-09 | TNFRSF19 |
| 3 | 11 | 2.67-2.71 | 3 | 2685080 | T/A | 2.75E-09 | MIPEP |
| 3 | 11 | 3.02-3.06 | 3 | 3039333 | A/G | 2.75E-09 |  |
| 3 | 11 | 3.16-3.20 | 3 | 3179238 | C/G | 2.75E-09 | MTMR6 |
| 3 | 11 | 2.75-2.79 | 8 | 2769518 | G/A | 2.8E-09 |  |
| 3 | 11 | 3.12-3.16 | 1 | 3141826 | C/A | 2.8E-09 | AMER2 |
| 3 | 11 | 2.70-2.74 | 1 | 2716177 | A/G | 2.86E-09 |  |
| 3 | 11 | 3.25-3.29 | 6 | 3270498 | C/T | 2.96E-09 | NUP58/ATP8A2 |
| 3 | 11 | 0.08-0.12 | 1 | 103293 | A/T | 3.51E-09 | ATP12A |
| 3 | 11 | 1.28-1.32 | 3 | 1309052 | A/G | 3.68E-09 | SAP18/SKA3 |
| 3 | 11 | 3.58-3.62 | 4 | 3598116 | A/G | 4.14E-09 |  |
| 3 | 11 | 4.50-4.54 | 3 | 4524983 | A/G | 4.43E-09 |  |
| 3 | 11 | 7.07-7.11 | 2 | 7088972 | T/C | 6.15E-08 |  |
| 3 | 11 | 3.74-3.78 | 1 | 3755009 | A/G | 8.99E-08 |  |
| 3 | 11 | 3.92-3.96 | 9 | 3937286 | C/T | 9.57E-08 |  |
| 3 | 11 | 66.48-66.52 | 1 | 66504100 | G/T | 1.05E-07 | MBNL2 |
| 3 | 11 | 5.90-5.94 | 1 | 5923683 | T/C | 1.35E-07 |  |
| 3 | 11 | 5.70-5.74 | 3 | 5724158 | T/C | 1.51E-07 |  |
| 3 | 11 | 3.68-3.72 | 6 | 3701087 | T/C | 1.68E-07 |  |
| 3 | 11 | 6.80-6.84 | 2 | 6824045 | A/T | 1.68E-07 |  |
| 3 | 11 | 3.62-3.66 | 3 | 3644293 | C/T | 1.73E-07 |  |
| 3 | 11 | 3.42-3.46 | 5 | 3440801 | C/T | 1.83E-07 |  |
| 3 | 11 | 3.52-3.56 | 3 | 3540246 | A/G | 1.83E-07 |  |
| 3 | 11 | 4.17-4.21 | 2 | 4191638 | T/C | 1.83E-07 |  |
| 3 | 12 | 27.37-27.41 | 1 | 27389620 | C/A | 5.4E-08 | SPAG9 |
| 3 | 13 | 108.10-108.14 | 1 | 108117987 | A/C | 6.4E-08 |  |
| 3 | 13 | 59.73-59.77 | 1 | 59748376 | A/T | 1.11E-07 | TRNT1/CRBN |
| 3 | 14 | 54.40-54.44 | 1 | 54424744 | A/C | 1.77E-09 |  |
| 3 | 14 | 142.99-143.03 | 1 | 143011389 | C/T | 1.23E-08 |  |
| 3 | 14 | 132.94-132.98 | 1 | 132955603 | C/A | 2.48E-08 |  |
| 3 | 14 | 78.19-78.23 | 1 | 78207439 | T/A | 1.01E-07 |  |
| 3 | 15 | 111.05-111.09 | 1 | 111065476 | G/A | 1.89E-09 | PLEKHM3 |
| 3 | 15 | 101.90-101.94 | 1 | 101917875 | C/A | 3.15E-09 | PLCL1 |
| 3 | 15 | 71.48-71.52 | 1 | 71500427 | C/A | 4.03E-08 | COBLL1 |
| 3 | 15 | 63.95-63.99 | 1 | 63967449 | C/T | 5.48E-08 |  |
| 3 | 15 | 140.92-140.96 | 1 | 140939604 | T/A | 1.14E-07 |  |
| 3 | 15 | 87.60-87.64 | 1 | 87615286 | G/T | 1.55E-07 | PPP1R1C/PDE1A |
| 3 | 16 | 82.28-82.32 | 1 | 82299037 | G/T | 4.09E-08 |  |
| 3 | 16 | 0.49-0.53 | 1 | 514594 | A/C | 1.35E-07 | CTNND2 |
| 3 | 17 | 11.32-11.36 | 1 | 11342154 | G/A | 1.49E-11 | IKBKB/POLB |
| 3 | 17 | 52.43-52.47 | 1 | 52450038 | A/C | 9.61E-10 | KCNG1 |
| 3 | 17 | 5.28-5.32 | 1 | 5295043 | C/A | 1E-09 | PDGFRL |
| 3 | 17 | 13.05-13.09 | 1 | 13067799 | C/A | 1.47E-09 | PSD3 |
| 3 | 17 | 69.66-69.70 | 1 | 69675942 | G/T | 1.27E-07 |  |
| 3 | 18 | 34.56-34.60 | 1 | 34579571 | A/C | 3.72E-09 |  |
| 3 | 18 | 40.52-40.56 | 1 | 40538768 | A/C | 7.82E-08 | RP9/FKBP9 |
| 3 | 23 | 89.29-89.33 | 1 | 89308525 | A/C | 3.19E-12 |  |
| 3 | 23 | 98.12-98.16 | 1 | 98138113 | T/C | 6.52E-10 | SOWAHD |
| 3 | 23 | 98.22-98.26 | 1 | 98237782 | A/T | 9.35E-09 | NKAP/NDUFA1 |
| 3 | 23 | 137.70-137.74 | 1 | 137720820 | G/T | 1.72E-08 |  |
| 3 | 23 | 69.58-69.62 | 4 | 69596000 | G/A | 1.41E-07 |  |
| 3 | 23 | 9.72-9.76 | 1 | 9737663 | A/C | 1.96E-07 |  |
| 3 | GL896387.1 | 0.01-0.05 | 1 | 28021 | AA/C | 1.93E-07 |  |
| 4 | 1 | 157.81-157.85 | 1 | 157829700 | T/A | 4.04E-08 | SERPINB8/SERPINB10 |
| 4 | 1 | 49.70-49.74 | 1 | 49723526 | G/T | 4.84E-08 |  |
| 4 | 3 | 14.65-14.69 | 1 | 14672610 | T/C | 2.08E-08 |  |
| 4 | 5 | 23.49-23.53 | 1 | 23509797 | G/A | 2.33E-08 |  |
| 4 | 9 | 7.84-7.88 | 1 | 7861339 | T/G | 5.51E-08 | RELT/FAM168A |

Table S2. Summary of significant chromosome regions including genomic level (0.05/N) for NM in Large White population, N is the number of SNPs used for analyses.

| Parity | SSC | Range (Mb) | SNP Number | Top SNP Position(bp) | Top SNP Allele | Top SNP P Value | Candidate Gene |
| --- | --- | --- | --- | --- | --- | --- | --- |
| 1 | 3 | 98.51-98.55 | 1 | 98528876 | G/A | 2.46E-08 |  |
| 1 | 6 | 7.64-7.68 | 1 | 7656724 | C/A | 3.76E-10 | DYNLRB2 |
| 1 | 15 | 53.17-53.21 | 1 | 53194098 | C/T | 6.65E-08 |  |
| 2 | 1 | 185.49-185.53 | 3 | 185506783 | C/A | 1.04E-07 |  |
| 2 | 1 | 32.30-32.34 | 2 | 32322537 | A/G | 1.1E-07 | AKAP7 |
| 2 | 4 | 132.34-132.38 | 1 | 132360260 | C/G | 1.25E-07 |  |
| 2 | 4 | 132.42-132.46 | 1 | 132444850 | G/A | 1.34E-07 |  |
| 2 | 5 | 65.60-65.64 | 1 | 65617114 | T/A | 1.44E-07 |  |
| 2 | 12 | 53.94-53.98 | 1 | 53960368 | C/T | 1.57E-08 | PIK3R6/PIK3R5 |
| 3 | 1 | 46.01-46.05 | 1 | 46027106 | G/T | 3.81E-09 |  |
| 3 | 1 | 126.23-126.27 | 1 | 126250077 | T/A | 1.19E-07 | SLC30A4 |
| 3 | 2 | 15.69-15.73 | 1 | 15706287 | G/T | 5.32E-17 | CKAP5 |
| 3 | 2 | 15.75-15.79 | 1 | 15773890 | A/G | 5.32E-17 | F2 |
| 3 | 2 | 159.31-159.35 | 6 | 159334109 | C/T | 9.58E-14 |  |
| 3 | 2 | 159.12-159.16 | 4 | 159142342 | C/T | 9.1E-12 |  |
| 3 | 2 | 159.28-159.32 | 3 | 159295635 | A/C | 3.43E-11 |  |
| 3 | 6 | 106.03-106.07 | 1 | 106053137 | A/T | 1.73E-08 |  |
| 3 | 6 | 106.03-106.07 | 1 | 106053141 | G/C | 1.73E-08 | THOC1 |
| 3 | 6 | 101.79-101.83 | 1 | 101813105 | A/C | 1.3E-07 |  |
| 3 | 7 | 119.10-119.14 | 1 | 119122551 | G/A | 1.79E-07 |  |
| 3 | 8 | 41.80-41.84 | 1 | 41820138 | C/T | 1.2E-07 | KDR |
| 3 | 10 | 65.58-65.62 | 1 | 65597724 | G/T | 4.64E-08 | AKR1C1 |
| 3 | 10 | 54.45-54.49 | 1 | 54466908 | A/T | 1.7E-07 | PLXDC2 |
| 3 | 11 | 64.03-64.07 | 1 | 64047767 | A/G | 1.4E-08 |  |
| 3 | 12 | 57.60-57.64 | 1 | 57618649 | T/G | 3.77E-08 |  |
| 3 | 18 | 15.29-15.33 | 5 | 15306205 | A/G | 1.05E-11 | EXOC4 |
| 3 | 18 | 15.38-15.42 | 3 | 15395120 | A/G | 5.72E-08 |  |
| 3 | 23 | 130.28-130.32 | 1 | 130304550 | G/T | 1.39E-07 |  |
| 3 | GL894691.2 | 0.07-0.11 | 1 | 86895 | T/C | 2.26E-08 |  |
| 4 | 1 | 278.42-278.46 | 1 | 278435455 | A/C | 2.15E-09 |  |
| 4 | 1 | 292.84-292.88 | 1 | 292861324 | A/C | 3.5E-08 |  |
| 4 | 1 | 208.02-208.06 | 1 | 208039823 | A/C | 9.94E-08 |  |
| 4 | 2 | 53.53-53.57 | 5 | 53550664 | A/T | 8.13E-08 |  |
| 4 | 5 | 51.17-51.21 | 1 | 51194350 | T/C | 1.23E-08 | ST8SIA1 |
| 4 | 5 | 53.84-53.88 | 1 | 53862342 | T/C | 1.43E-08 | PLEKHA5 |
| 4 | 5 | 51.77-51.81 | 4 | 51793209 | T/G | 1.84E-08 |  |
| 4 | 5 | 50.24-50.28 | 2 | 50259094 | A/T | 2.01E-08 |  |
| 4 | 5 | 51.90-51.94 | 8 | 51920412 | A/G | 2.58E-08 | GYS2/SPX/GOLT1B |
| 4 | 5 | 49.74-49.78 | 2 | 49763855 | A/G | 2.89E-08 | SOX5 |
| 4 | 5 | 52.70-52.74 | 2 | 52719540 | T/C | 3.52E-08 |  |
| 4 | 5 | 53.69-53.73 | 1 | 53705159 | T/C | 3.95E-08 |  |
| 4 | 5 | 53.04-53.08 | 1 | 53064907 | A/G | 3.99E-08 |  |
| 4 | 5 | 50.58-50.62 | 1 | 50596863 | A/G | 4.25E-08 |  |
| 4 | 5 | 51.68-51.72 | 5 | 51702429 | C/T | 4.27E-08 | KCNJ8/ABCC9 |
| 4 | 5 | 36.73-36.77 | 5 | 36749866 | T/G | 8.5E-08 |  |
| 4 | 7 | 116.70-116.74 | 2 | 116719034 | G/T | 1.32E-10 | SYNE3 |
| 4 | 15 | 62.98-63.02 | 14 | 63000645 | A/T | 9.45E-10 |  |
| 4 | 17 | 47.69-47.73 | 1 | 47709929 | T/G | 1.42E-10 | TP53TG5/SYS1 |
| 4 | GL894238.2 | -0.04 | 1 | 1111 | T/G | 2.83E-08 |  |
| 5 | 2 | 14.95-14.99 | 7 | 14966625 | G/A | 1.06E-09 | AGBL2/MTCH2 |
| 5 | 2 | 52.21-52.25 | 2 | 52227328 | T/C | 2.76E-09 | LOC100525099 |
| 5 | 2 | 40.07-40.11 | 1 | 40089863 | C/A | 2.26E-08 |  |
| 5 | 2 | 11.48-11.52 | 1 | 11501415 | G/T | 2.33E-08 |  |
| 5 | 2 | 147.73-147.77 | 1 | 147750147 | A/G | 5.05E-08 |  |
| 5 | 2 | 100.22-100.26 | 1 | 100240077 | T/C | 5.73E-08 |  |
| 5 | 3 | 27.64-27.68 | 2 | 27657943 | T/A | 1.68E-08 |  |
| 5 | 3 | 27.86-27.90 | 2 | 27881765 | G/C | 1.68E-08 |  |
| 5 | 4 | 62.24-62.28 | 2 | 62259407 | A/C | 6.18E-13 | STAU2 |
| 5 | 4 | 21.46-21.50 | 1 | 21476637 | A/T | 5.71E-09 |  |
| 5 | 4 | 13.08-13.12 | 1 | 13098579 | A/G | 8.15E-09 |  |
| 5 | 4 | 62.31-62.35 | 1 | 62329223 | A/G | 2.23E-08 |  |
| 5 | 4 | 106.16-106.20 | 1 | 106184889 | T/G | 2.28E-08 | TRIM33 |
| 5 | 4 | 53.63-53.67 | 1 | 53650496 | A/C | 2.71E-08 |  |
| 5 | 6 | 68.70-68.74 | 1 | 68723593 | A/C | 8.1E-11 |  |
| 5 | 6 | 43.25-43.29 | 1 | 43271313 | A/G | 6.88E-09 | PEPD |
| 5 | 6 | 137.87-137.91 | 1 | 137887738 | G/T | 1.84E-07 | SLC44A5 |
| 5 | 7 | 121.12-121.16 | 5 | 121138988 | C/G | 2.16E-08 | YY1 |
| 5 | 8 | 74.61-74.65 | 1 | 74627749 | T/C | 1.67E-08 | PLRG1 |
| 5 | 8 | 68.15-68.19 | 1 | 68169820 | T/A | 4.36E-08 | SLC4A4 |
| 5 | 8 | 5.54-5.58 | 2 | 5558816 | A/G | 6.84E-08 |  |
| 5 | 8 | 140.87-140.91 | 1 | 140892875 | A/G | 1.4E-07 |  |
| 5 | 9 | 16.43-16.47 | 1 | 16446768 | T/C | 1.6E-09 |  |
| 5 | 9 | 10.43-10.47 | 1 | 10452963 | T/C | 1.76E-08 |  |
| 5 | 10 | 21.60-21.64 | 3 | 21623235 | C/T | 3.25E-15 |  |
| 5 | 10 | 50.55-50.59 | 1 | 50568571 | G/A | 2.88E-10 | ARHGAP21 |
| 5 | 10 | 34.46-34.50 | 1 | 34477569 | A/C | 8.9E-08 |  |
| 5 | 12 | 37.49-37.53 | 1 | 37512908 | A/G | 2.8E-09 | PPM1D |
| 5 | 12 | 55.96-56.00 | 1 | 55979598 | G/A | 1.08E-08 |  |
| 5 | 13 | 110.81-110.85 | 1 | 110833752 | C/T | 2.26E-08 | FNDC3B |
| 5 | 13 | 120.98-121.02 | 1 | 120996941 | A/C | 4.26E-08 | MCCC1 |
| 5 | 13 | 93.07-93.11 | 1 | 93090375 | A/T | 5.42E-08 |  |
| 5 | 13 | 87.43-87.47 | 1 | 87452441 | A/C | 6.27E-08 |  |
| 5 | 13 | 50.05-50.09 | 1 | 50069499 | A/T | 1.25E-07 |  |
| 5 | 14 | 80.64-80.68 | 1 | 80664055 | T/C | 2.08E-11 |  |
| 5 | 14 | 14.88-14.92 | 1 | 14897752 | G/T | 9.98E-10 | GATA4 |
| 5 | 14 | 40.61-40.65 | 1 | 40626506 | G/T | 1.71E-08 | ACADS/MLEC/UNC119B |
| 5 | 15 | 73.91-73.95 | 1 | 73929378 | G/T | 4.14E-13 |  |
| 5 | 15 | 52.89-52.93 | 4 | 52912314 | T/C | 1.44E-09 |  |
| 5 | 15 | 51.16-51.20 | 1 | 51175162 | T/C | 1.73E-08 |  |
| 5 | 16 | 48.96-49.00 | 1 | 48979292 | T/G | 7.55E-14 | ZNF366 |
| 5 | 17 | 50.86-50.90 | 2 | 50880718 | T/G | 2.49E-10 | STAU1 |
| 5 | 17 | 13.65-13.69 | 1 | 13672792 | T/G | 6.62E-08 |  |
| 5 | 17 | 63.05-63.09 | 1 | 63066086 | A/C | 1.09E-07 | PCMTD2 |
| 5 | 17 | 8.27-8.31 | 1 | 8289034 | G/T | 1.29E-07 |  |
| 5 | 18 | 0.33-0.37 | 1 | 348091 | T/G | 7.31E-11 |  |
| 5 | 23 | 36.37-36.41 | 1 | 36392108 | T/G | 2.77E-08 | MED14 |

Table S3. Summary of significant chromosome regions including genomic level (0.05/N) for NS in Landrace population, N is the number of SNPs used for analyses.

| Parity | SSC | Range (Mb) | SNP Number | Top SNP Position(bp) | Top SNP Allele | Top SNP P Value | Candidate Gene |
| --- | --- | --- | --- | --- | --- | --- | --- |
| 1 | 1 | 26.52-26.56 | 1 | 26543276 | T/A | 1.17E-08 |  |
| 2 | 15 | 147.19-147.23 | 1 | 147205892 | T/G | 3.79E-10 |  |
| 3 | 1 | 85.70-85.74 | 1 | 85721479 | T/C | 4.7E-08 |  |
| 3 | 1 | 276.16-276.20 | 1 | 276178510 | A/G | 1.59E-07 |  |
| 3 | 2 | 12.48-12.52 | 1 | 12497701 | T/A | 1.24E-07 | ZFP91 |
| 3 | 3 | 129.06-129.10 | 1 | 129076023 | T/C | 2.27E-08 |  |
| 3 | 3 | 131.82-131.86 | 3 | 131841442 | G/A | 1.29E-07 |  |
| 3 | 4 | 73.53-73.57 | 1 | 73553176 | T/G | 8.16E-10 |  |

Table S4. Summary of significant chromosome regions including genomic level (0.05/N) for NS in Large White population, N is the number of SNPs used for analyses.

| Parity | SSC | Range (Mb) | SNP Number | Top SNP Position(bp) | Top SNP Allele | Top SNP P Value | Candidate Gene |
| --- | --- | --- | --- | --- | --- | --- | --- |
| 2 | 9 | 118.97-119.01 | 5 | 118987531 | G/A | 8.89E-08 | ASTN1/BRINP2 |
| 5 | 11 | 16.77-16.81 | 1 | 16788784 | A/C | 6.28E-09 |  |
